# Genetic Transformation of Recalcitrant Cassava by Embryo Selection and Increased Hormone Levels

**DOI:** 10.3390/mps1040042

**Published:** 2018-11-13

**Authors:** Ezequiel Matías Lentz, Sabrina Eisner, Emily Jane McCallum, Kim Schlegel, Francisco de Assis de Paiva Campos, Wilhelm Gruissem, Hervé Vanderschuren

**Affiliations:** 1 Department of Biology, Plant Biotechnology, ETH Zurich, 8092 Zürich, Switzerland; ezequiel.lentz@gmail.com (E.M.L.); saeisner@ethz.ch (S.E.); emilym@ethz.ch (E.J.M.); kims@ethz.ch (K.S.); wgruisse@ethz.ch (W.G.); 2 Department of Biochemistry and Molecular Biology, Universidade Federal do Ceará, 60455-900 Fortaleza, Brazil; bioplant@ufc.br; 3 Plant Genetics, TERRA Teaching and Research Center, University of Liège, Passage des Déportés 2, 5030 Gembloux, Belgium

**Keywords:** cassava, somatic embryo, FEC, transformation, *Agrobacterium*, farmer-preferred cultivar

## Abstract

Genetic engineering is considered to be an important tool for the improvement of cassava. Cassava is a highly heterozygous crop species for which conventional breeding is a lengthy and tedious process. Robust transformation is based on Agrobacterium-mediated transformation of friable embryogenic callus (FEC). Production of FEC is genotype-dependent and considered to be a major bottleneck for the genetic transformation of cassava. As a consequence, routine genetic transformation has only been established for a handful of cassava cultivars. Therefore, development of procedures enabling efficient production of high-quality cassava FEC is required to allow the translation of research from the model cultivar to farmer-preferred cassava cultivars. Here we study the FEC production capacity of Brazilian cassava cultivars and report the modification of the protocol for the genetic transformation of Verdinha (BRS 222), a recalcitrant cultivar with high potential for protein production that is extensively used by farmers in Brazil.

## 1. Introduction

Cassava (*Manihot esculenta* Crantz) is a major crop for the local economy in Northeast Brazil. In the Coastal Tableland of Northeast Brazil, cassava production is severely constrained by drought and pests [1,2,3]. Although cassava varieties are conventionally bred in Brazil [2], cassava breeding remains a tedious and lengthy task [4,5]. Therefore, genetic transformation offers the possibility to ease and speed up the generation of cassava cultivars with improved traits [6]. Recent progress in cassava genome editing to generate lines with improved virus resistance and modified starch have relied on stable genetic transformation [7,8].

Several parameters, such as the type of explants, plant growth regulators, antibiotic selection, light quality, gene transfer approaches and plant regeneration methods play important roles in the genetic transformation of plants [9]. However, factors associated with plant tissue growth and regeneration influence to a great extent the transformation efficiency [10], and precise adjustments of media and hormone concentrations can result in successfully transformed recalcitrant cultivars [11]. The genetic transformation of cassava was first reported twenty years ago [12,13]. The initially reported methods included the transformation of friable embryogenic callus (FEC) using the gun-mediated delivery of DNA and the *Agrobacterium*-mediated transformation of cassava cotyledons. These approaches were based on somatic embryogenesis and shoot organogenesis, respectively [6]. Over the last two decades, the *Agrobacterium*-mediated transformation of FEC has emerged as the most suitable method. This is because it has a relatively high efficiency, and the production of chimeric tissues is limited compared to other methods [6,14]. Following the establishment of a robust method for genetic transformation of the cassava model cultivar 60444 [14,15,16], the improved protocol proved to be also suitable for the transformation of recalcitrant farmer-preferred landraces and varieties from Africa and Asia [17,18,19]. Importantly, the improved protocol appeared to be suitable to its establishment in independent laboratories in Africa [18,19,20]. However, the main obstacle to translational research in cassava remains the production of cassava tissues suitable for genetic transformation and the regeneration of transgenic lines. The FEC induction and multiplication steps appear to be particularly problematic and often require protocols with substantial modifications to achieve the production of FEC amenable for genetic transformation and regeneration of transgenic plantlets [17,19,21,22].

The cultivars Verdinha (BRS 222), Amansa burro (BRS 293) and Tapioqueira (BRS 325) were developed and released by EMBRAPA. They are adapted to Coastal Tableland of Northeast Brazil with traits such as drought tolerance [3,23]. The Verdinha cultivar has also been reported to have a good potential for protein production [24]. In the present study, we investigated the capacity and amenability of Verdinha, Amansa burro and Tapioqueira for FEC production and genetic transformation.

## 2. Materials and Methods

### 2.1. Plant Material and Genetic Constructs

Stem cuttings of Verdinha BRS 222, Amansa burro BRS 293 and Tapioqueira BRS 325 were obtained from a cassava field collection (Universidade Federal do Ceará, Fortaleza, Brazil). Stem cuttings were disinfected and transferred to in vitro culture as indicated by Feitosa et al. [25]. In vitro plantlets, including the model cultivar 60444, were propagated on cassava basic media (CBM) [14] in growth cabinet at 28 °C (16 h light/8 h dark). The plasmid pCambia 1305.1 (Genbank: AF354045.1) containing the GUSPlus reporter gene coding for the β-glucuronidase (GUS) and the hygromycin resistance gene for plant selection, was used for the genetic transformation experiments. Three independent transformation experiments were performed for the Verdinha BRS 222 cultivar and the model cultivar 60444 with pCambia 1300-based constructs.

### 2.2. Friable Embryogenic Callus Induction, Maintenance and Regeneration Test

FEC induction was performed according to the standard protocol described by Bull et al. [14], using axillary buds from in vitro plantlet material. FECs were subcultured every two weeks in Gresshoff & Doy (GD, [26]) plates. For the regeneration test, FEC clumps were incubated on somatic embryo emerging medium (MSN) containing 250 mg/L carbenicillin (C250) as indicated for the “FEC viability assay” by Bull et al. [14]. The adapted protocol included modifications in the cyclic embryogenesis step and in the picloram concentration of GD plates for the maintenance and multiplication of FEC.

### 2.3. Genetic Transformation

*Agrobacterium*-mediated transformation experiments were performed with the FEC from Verdinha (BRS 222) following the method described by Bull et al. [14] using the LBA4404 *Agrobacterium* strain. Regenerated plants were identified as putative transgenic lines by the hygromycin-rooting test: a stem cutting of the in vitro plantlet was transferred to a jar containing Cassava Basic Medium (CBM) including 50 mg/L carbenicillin (C50) and 10 mg/L hygromycin (H10), and the development of roots indicated the presence of the selection marker. Rooting test-positive lines were selected for further characterization.

### 2.4. Molecular Characterization of Transgenic Plants and β-Glucuronidase Staining

Leaf genomic DNA for PCR and Southern blot was isolated according to Soni and Murray [27]. PCR detection of the GUS reporter gene was performed with primers 5′-GTGGTACCCTGGATCGCGAAAACTGTGGAATTG-3′ and 5′-GTACTAGTGCATTACGCTGCGATGGATTCCGG-3′. Southern blot analysis was conducted as described by Vanderschuren et al. [28], using a digoxigenin (DIG)-labeled HptII probe on XbaI-treated DNA samples. In vitro whole plantlets were GUS-stained and photographed as indicated in Lentz et al. [29].

### 2.5. Statistical Analysis

For the comparison of somatic embryo (SE) structure, three samples consisting in 108, 185 and 261 clusters of SEs obtained with the standard protocol were compared with three samples of 226, 259 and 236 SEs obtained with the adapted protocol. The distribution of SEs among globular, torpedo and cotyledonary structures was determined by observation with a dissecting microscope, and these values were analyzed with two-tailed Student’s *t*-tests. The SEs structure distribution among the different cultivars was analyzed with the Tukey’s multiple comparison test. The induction efficiency was calculated with the values obtained from three independent FEC induction assays performed with the model cultivar 60444 (standard protocol), BRS 222 (standard protocol) and BRS 222 using the adapted protocol. These three groups were analyzed by a Tukey’s multiple comparison test. The transformation efficiency of the model cultivar 60444 and Verdinha BRS 222 in three independent assays with pCambia 1300-based plasmids, were analyzed with two-tailed *t*-tests. The software employed was MATLAB R2016a (MathWorks Inc., MA, USA).

### 2.6. Experimental Design

A first experiment consisted of SEs production using the selected Brazilian cultivars and the model accession cv. 60444 (Figure S1). Generated SEs were employed in the first FEC induction attempt including three groups of SEs from each cultivar as indicated in Table S1, following the standard protocol. Three additional FEC induction experiments with the cultivar Verdinha BRS 222 were conducted as indicated in Table 1, following the adapted protocol. The BRS 222 FEC batch obtained from the first induction with the adapted protocol was further employed to (1) study the impact of picloram concentration on multiplication rate and (2) to perform three independent genetic transformations with pCambia-based vectors.

## 3. Results

### 3.1. Friable Embryogenic Callus Induction

Axillary buds from all three cultivars (Verdinha, Amansa burro and Tapioqueira) produced somatic embryos (SEs) on callus induction medium (CIM) (Figure S1). Amansa burro was previously shown to display moderate levels of somatic embryogenesis [25]. In the present study, SEs of good quality were observed for the three cultivars, which were subsequently transferred to GD medium for FEC induction. The model cultivar 60444 was included as positive control and for comparison of FEC production. While the cultivar 60444 produced FEC with an efficiency of 50%, none of the Brazilian cultivars produced FEC with the standard induction protocol [14] (Table S1, Figure S1). Observation of SEs present after four passages on CIM and prior to transfer to the FEC induction media (GD) revealed a significantly higher percentage (68%) of SEs with torpedo structure in the model cultivar 60444 compared to the Brazilian cultivars (Table S1). Embryos with torpedo structure could only be observed for Tapioqueira and Verdinha cultivars, however at very low frequencies (1–2%) (Table S1). Previous studies using genotypes from the northeast of Brazil have shown that globular (“coral-like”) SEs initially develop on somatic embryo induction medium and that sequential multiplications lead to advanced developmental stages, first torpedo-(“finger-like”) and eventually cotyledonary-stages [25].

### 3.2. Friable Embryogenic Callus Induction with the Adapted Protocol

**Figure 1:**
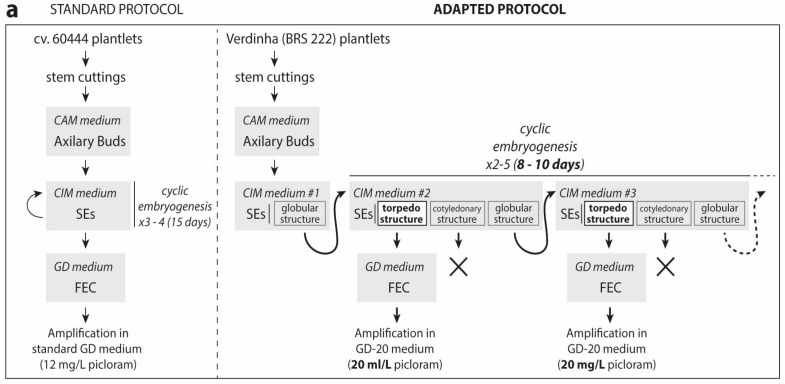

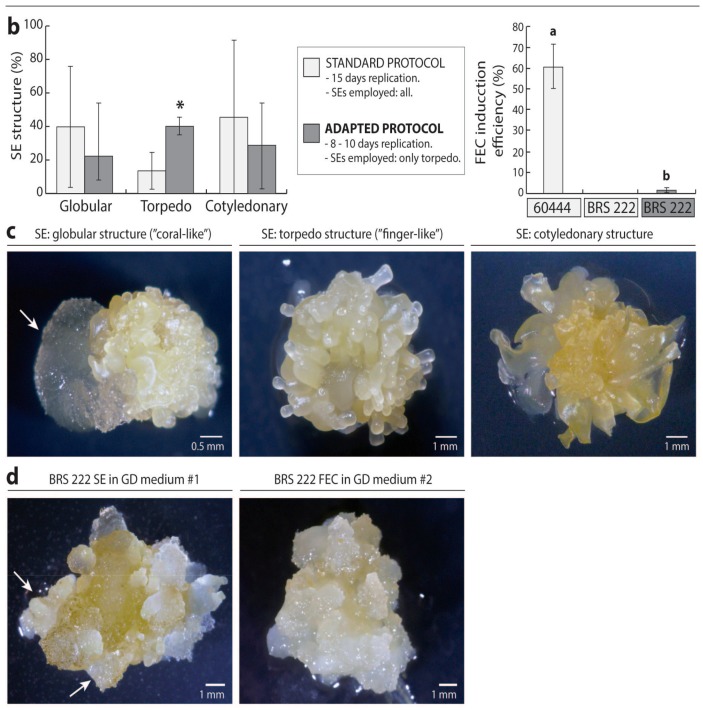
Adapted FEC (friable embryogenic callus) induction protocol for the Brazilian cultivar Verdinha (BRS 222). (**a**) Comparison of the standard and adapted protocol. (**b**) SEs (somatic embryo) structure distribution (Verdinha) during successive replications on CIM (callus induction medium) plates. Left: asterisk indicates a significant difference (*p* < 0.05) in a two-tailed *t* test. Right: Tukey’s multiple comparison test (*p* < 0.01). (**c**) Examples of Verdinha SEs with globular (“coral-like”), torpedo (“finger-like”) and cotyledonary structure. The white arrow indicates non-embryogenic friable calli (NEFC) material (scale bars = 1 mm). (**d**) Selection of putative FEC (arrows) from GD (Gresshoff & Doy) plate #1 (to be transferred to GD plate #2) (scale bars = 1 mm). CAM: cassava axillary medium.

Compared to the model cv. 60444, Verdinha showed a significantly smaller percentage of SEs with torpedo structure, and this percentage was even smaller for the other Brazilian cultivars (Table S1). Therefore, we modified the protocol in order to increase the percentage of embryos at the torpedo stage (Figure 1). The torpedo SEs from Verdinha appeared after two cycles on CIM. We observed that shortening the multiplication time of SEs on CIM from 15 days to 8–10 days increased the ratio of torpedo-stage over cotyledonary-stage SEs (Figure 1b,c). At each SEs multiplication step, torpedo-stage SEs were transferred to GD medium for FEC induction. SEs showing cotyledonary-stage were discarded. As anticipated, the use of torpedo-stage SEs allowed a very low but reproducible FEC induction for Verdinha (BRS 222) (Table 1). The selection of potential FEC emerging from SEs on the first induction medium (GD #1) was particularly challenging, and as a general rule any globular putative FEC material was transferred to GD medium #2 (Figure 1d). Further replication of selected putative FEC on GD medium was used to confirm the embryogenic nature of the selected tissues (Figure S2a).

During the multiplication of Verdinha FEC, we observed spontaneous FEC regeneration on standard GD medium (Figure S2a). This phenomenon made the selection and maintenance of Verdinha FEC particularly challenging. Importantly, FEC regeneration occurred under hygromycin selection (Figure S2b), suggesting that the production of transgenic Verdinha plantlets using the standard FEC-based transformation protocol [12,14] could be problematic. Our initial attempt to transform Verdinha FEC led to the generation of 76 putative transgenic lines, which appeared to be escapees using the rooting test previously reported [12].

### 3.3. Increase of Hormone Concentration to Avoid Spontaneous Regeneration

**Figure 2:**
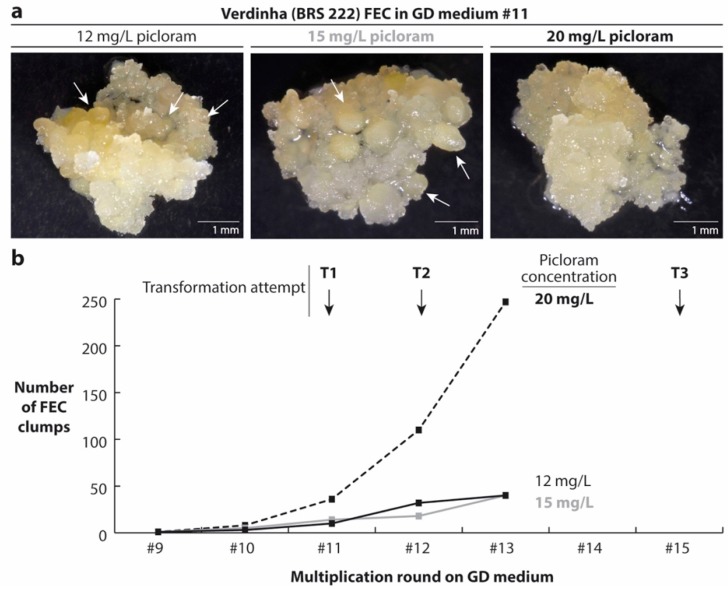
Effect of hormone concentrations on Verdinha FEC multiplication. (**a**) Representative pictures of FEC clumps grown on GD medium supplemented with 12 (standard), 15 and 20 mg/L of picloram. Regenerating FEC are indicated with arrows. Scale bars = 1 mm. (**b**) Multiplication curve of FEC under different picloram concentrations. T1, T2 and T3 represent three independent transformations carried out with the FEC material obtained from the replication round in GD medium #11, #12 and #15, respectively.

In order to reduce the development of mature embryos, we tested increasing picloram concentrations in GD medium for the multiplication of Verdinha FEC (Figure 2). Our results indicated that Verdinha FEC maintenance and multiplication was highly improved at the picloram concentration of 20 mg/L as compared to the standard concentration (12 mg/L). The higher concentration of picloram prevented the spontaneous maturation of Verdinha FEC observed in the standard GD medium (Figure 2a) and increased their multiplication rate (Figure 2b).

### 3.4. Agrobacterium-Mediated Transformation of BRS 222 Friable Embryogenic Callus 

**Figure 3:**
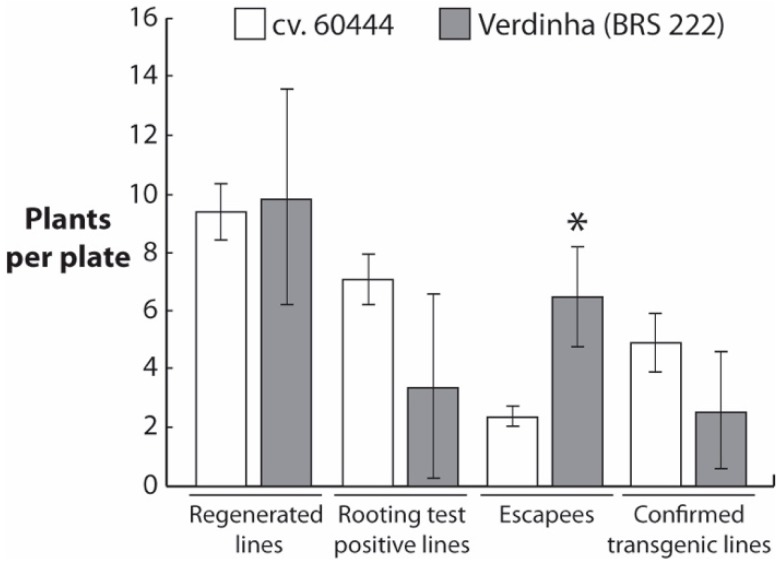
Comparison of transformation efficiency between the model genotype cv. 60444 and Verdinha (BRS 222). Three independent transformations with the pCambia 1300-based constructs are presented. Values are normalized according to the number of FEC plates used for transformation. Transgenic lines were confirmed by PCR and Southern blot. Asterisk indicates a significant difference (*p* < 0.05) in a two-tailed *t*-test.

Verdinha FEC material multiplied on the adapted GD medium containing 20 mg/L picloram were used to sequentially perform three independent *Agrobacterium*-mediated transformations (Figure 3, Table 2). Agrobacterium-mediated transformation of Verdinha FEC was performed with the binary vector pCambia 1305.1 (Genbank: AF354045.1) and selection of transgenic plantlets followed the procedure described by Bull et al. [14].

**Figure 4:**
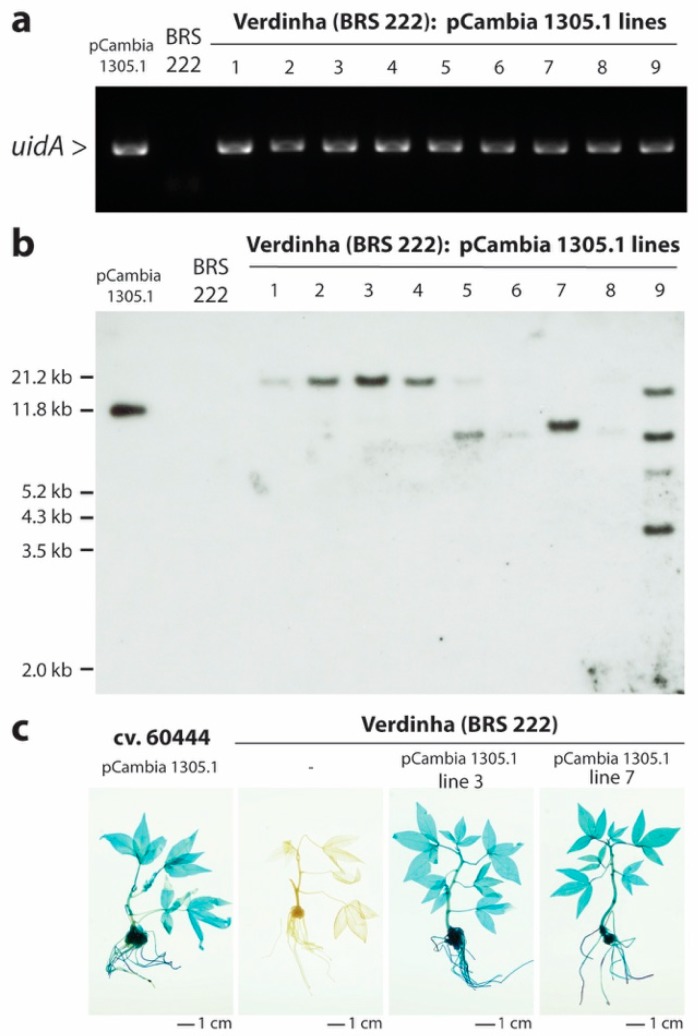
Summary of the molecular characterization of transgenic Verdinha (BRS222) plants. (**a**) PCR detection of the GUS transgene in 1% agarose/TAE gel, stained with EtBr. (**b**) Confirmation of transgene integration by Southern blot. XbaI-treated DNA samples were analyzed with a DIG-labeled HptII probe according to Vanderschuren et al. [28]. (**c**) β-glucuronidase expression analysis of in vitro plantlets according to Lentz et al. [29]. Scale bars = 1 cm.

Transgene presence, integration and GUS expression were confirmed by hygromycin-rooting test, PCR, Southern blot analysis and GUS staining, respectively (Figure 4, Figures S3 and S4). We could observe several independent transgenic events in Verdinha (Figure 3). In the Southern blot analysis, some of the characterized lines displayed faint and strong hybridization bands that could be due to incomplete digestion (Figure 4 and Figure S4). Selected transgenic Verdinha BRS 222 lines were subsequently grown under greenhouse conditions. The transgenic Verdinha BRS 222 lines displayed a phenotype that was undistinguishable from the control wild-type lines (Figure S5). These results indicate that the Brazilian farmer-preferred cultivar Verdinha (BRS 222) is amenable to genetic transformation, however at lower rate when compared to the model cv. 60444 and with significantly higher number of escapees (Figure 3).

## 4. Discussion

In conclusion, we successfully established an adapted protocol to transform the Brazilian farmer-preferred cassava cultivar Verdinha. We identified the production and maintenance of high quality FEC as the limiting factor to perform the standard Agrobacterium-mediated transformation of FEC. SEs at the torpedo-stage appeared to be suitable tissues to induce FEC formation from Verdinha cultivar. Although *Agrobacterium*-mediated transformation of FEC has emerged as the method of choice to produce transgenic cassava, production of FEC is often the limiting step as it is highly genotype-dependent [6,30]. Preliminary studies have been performed in order to identify molecular markers associated with FEC induction potential, in particular on embryos at the torpedo and cotyledonary-stage [31], but the identification of such markers has remained elusive. The present results indicate that torpedo-stage SEs can be obtained in higher abundance by shortening multiplication time on the SE inducing medium. Another torpedo-generating approach could focus on the use of plant hormone regulators or adjusted media [32,33,34,35] as suggested by recent studies with model and non-model farmer-preferred cassava cultivars [22,36]. The recent improvement of de novo shoot organogenesis from cassava also holds some promise for the development of genetic transformation methods that are not requiring the production of FEC [37]. However, the selection of torpedo-stage SEs for FEC induction combined with an increased concentration of picloram in GD medium appeared to be sufficient to enable genetic transformation and regeneration of transgenic Verdinha plantlets. Our adapted protocol provides an alternative approach to genetically transform cassava genotypes recalcitrant to FEC production and genetic transformation.

## Figures and Tables

**Table 1:** Friable embryogenic callus (FEC) induction of Verdinha (BRS 222) using the modified protocol.

FEC Induction	Starting Material: # of Explants (ABs)	# of Explants Inducing SEs	SEs Clusters Used for FEC Induction *	FEC Clumps Induced in GD Plates	FEC Induction Efficiency (%)
1	387	382	594	6	1
2	99	94	180	2	1
3	198	196	284	6	2

* Only somatic embryos (SEs) with torpedo structure were transferred to GD plates for FEC induction (SEs with cotyledonary structure were discarded and those with globular structure were replicated in callus induction medium (CIM) plates until the torpedo structure was observed and used for FEC induction). FEC: Friable embryogenic calli; ABs: axillary buds; SEs were subcultured on CIM (solid medium for induction of SEs) between 2–5 times. GD: solid medium for induction of FEC and maintenance. Each plate contained 9 ABs, SEs clusters or FEC clumps. Inductions 1, 2 and 3 are independent experiments.

**Table 2:** Production of transgenic Verdinha BRS 222 plants with the modified protocol.

Transformation Experiment	Regenerated Lines	Rooting-Test Positive Lines	Escapees	GUS-Expressing Lines	PCR- and Southern Blot-Confirmed Lines
I	35	1 (3%)	34 (97%)	1	1
II	14	7 (50%)	7 (50%)	7	7
III	28	13 (46%)	15 (54%)	13	13

## Supplementary Materials

The following are available online at http://www.mdpi.com/2409-9279/1/4/42/s1, Figure S1: FEC induction using the standard protocol. Table S1: FEC induction of cassava Brazilian cultivars with the standard protocol; Figure S2: Spontaneous maturation of embryos from Verdinha FEC; Figure S3: PCR and Southern blot analysis of transgenic Verdinha (BRS 222) plants; Figure S4: Phenotype of pCambia 1305.1-transgenic Verdinha (BRS 222) plants in greenhouse conditions.
