# Supplementary material for: Genetic Transformation of Recalcitrant Cassava by Embryo Selection and Increased Hormone Levels
Source: Methods Protoc. 2018 Nov 13;1(4):42. doi: 10.3390/mps1040042 (PMC6481083; doi:10.3390/mps1040042)

Figure S1. FEC induction using the standard protocol. The model genotype cv. 60444 was included as positive control. Representative pictures of Somatic Embryos (SEs), and putative Friable Embryogenic Calli (FEC) are shown for the Brazilian cultivars Verdinha (BRS 222), Amansa burro (BRS 293) and Tapioqueira (BRS 325). Representative pictures are shown for each cultivar, where CIM #1 and CIM #2 are first and second induction media, and GD #3 correspond the third FEC induction media (scale bars = 1 mm).

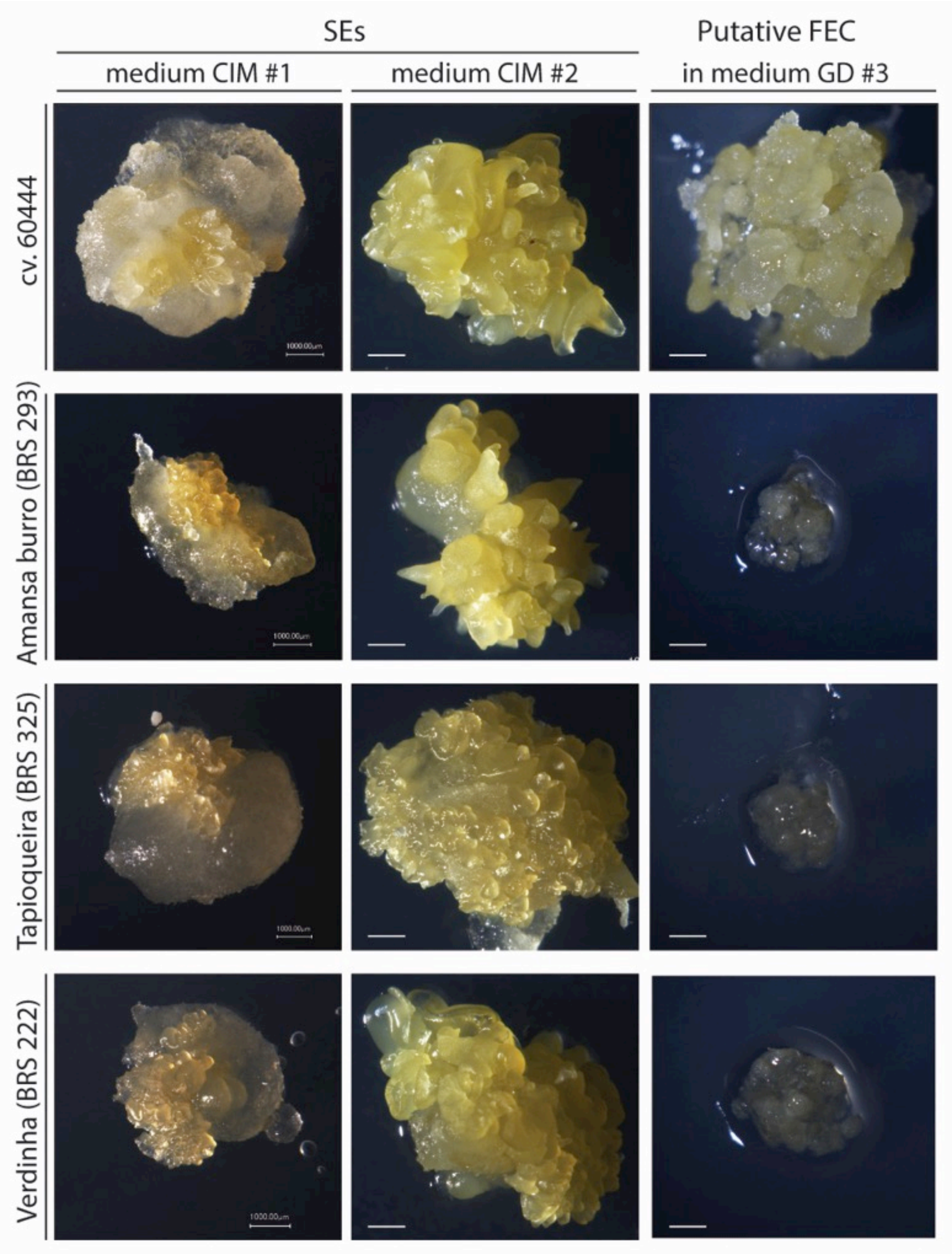

Table S1. FEC induction of cassava Brazilian cultivars Verdinha (BRS 222), Amansa burro (BRS 293) and Tapioqueira (BRS 325) (standard protocol, [9]). FEC: Friable Embryogenic Calli; CAM (Cassava Axillary Medium), the solid medium for enlargement of axillary buds (ABs); SEs were subcultured 4 times on CIM (Cassava Induction Medium), the solid medium for induction of SEs. GD: solid medium for induction and maintenance of FEC. Each plate contained up to 9 ABs, SEs clusters or FEC clumps.

FEC induction attempt	Cassava genotype	Starting material: CAM plates with ABs	Passages of SEs through CIM plates	Structure of selected SEs for FEC induction, transferred to GD plates	SEs clusters used for FEC induction	FEC clumps induced in GD plates	FEC induction efficiency (%)
**I**	cv. 60444	3	4	Cotyledonary (27%)<br>Globular (5%)<br>Torpedo (68%)	68	34	**50**
**I**	Amansa burro (BRS 293)	8	4	Cotyledonary (87%)<br>Globular (13%)	180	0	**0**
**I**	Tapioqueira (BRS 325)	13	4	Cotyledonary (78%)<br>Globular (21%)<br>Torpedo (1%)	279	0	**0**
**I**	Verdinha (BRS 222)	12	4	Cotyledonary (95%)<br>Globular (3%)<br>Torpedo (2%)	261	0	**0**

FEC: Friable Embryogenic Calli; CAM (Cassava Axillary Medium), the solid medium for enlargement of axillary buds (ABs); SEs were subcultured 4 times on CIM (Cassava Induction Medium), the solid medium for induction of SEs. GD: solid medium for induction and maintenance of FEC. Each plate contained up to 9 ABs, SEs clusters or FEC clumps.

Figure S2. Spontaneous regeneration of embryos from Verdinha FEC. **(a)** Examples of FEC clumps with regenerating embryos (arrows). **(b)** Regeneration test on embryo maturation medium (MSN) (C250: 250 mg/L carbenicillin; H15: 15 mg/L hygromycin; [9]). The arrow indicates an escape under hygromycin selection. Scale bars = 1 mm.

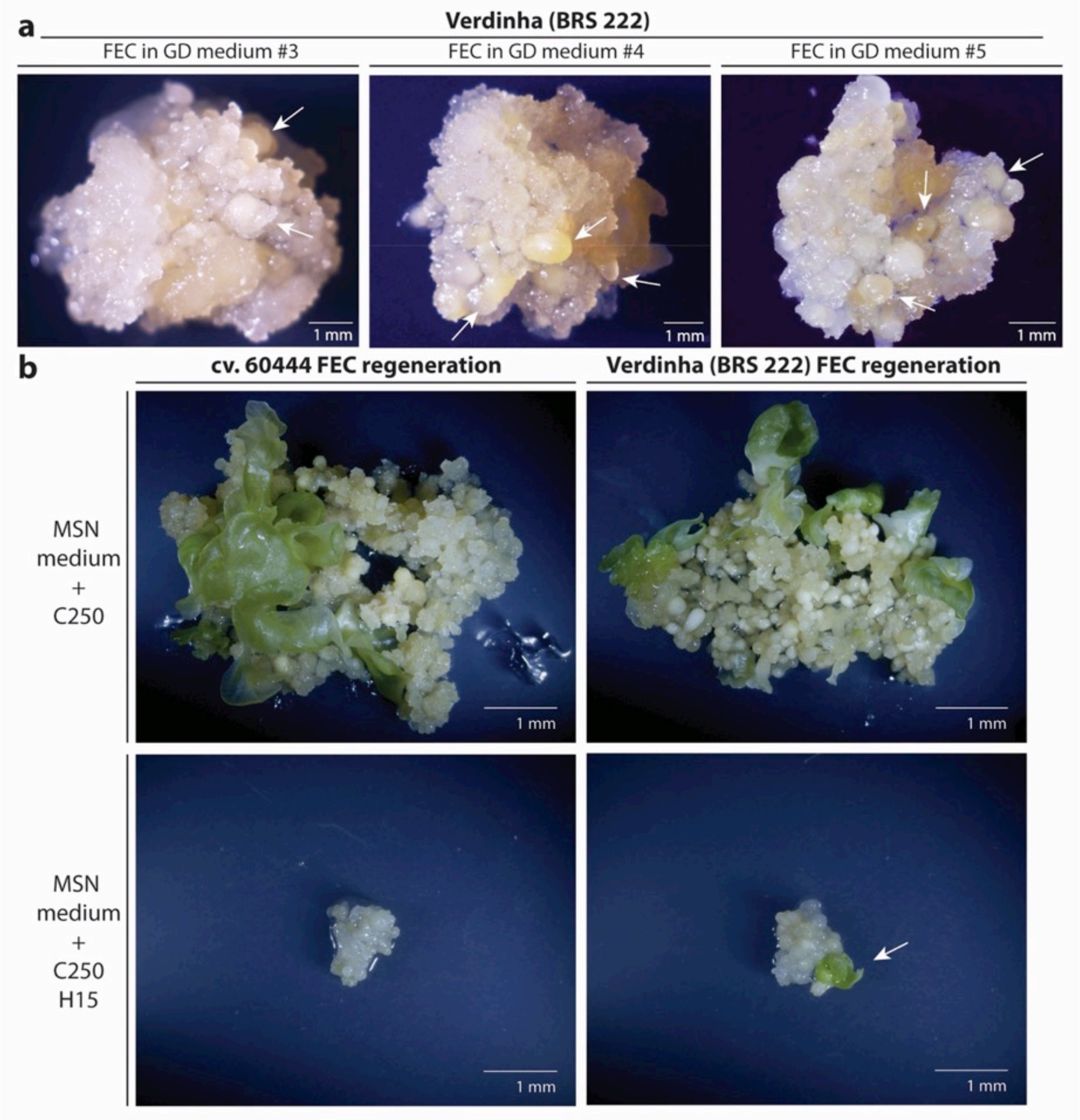

Figure S3 PCR and Southern blot analysis of transgenic Verdinha (BRS 222) plants. **(a)** PCR detection of the *uidA* transgene in a 1% agarosa/TAE gel stained with EtBr. **(b)** Confirmation of transgene integration by Southern blot. *Xba* I-treated DNA samples were analyzed with a DIG-labeled *HptII* probe.

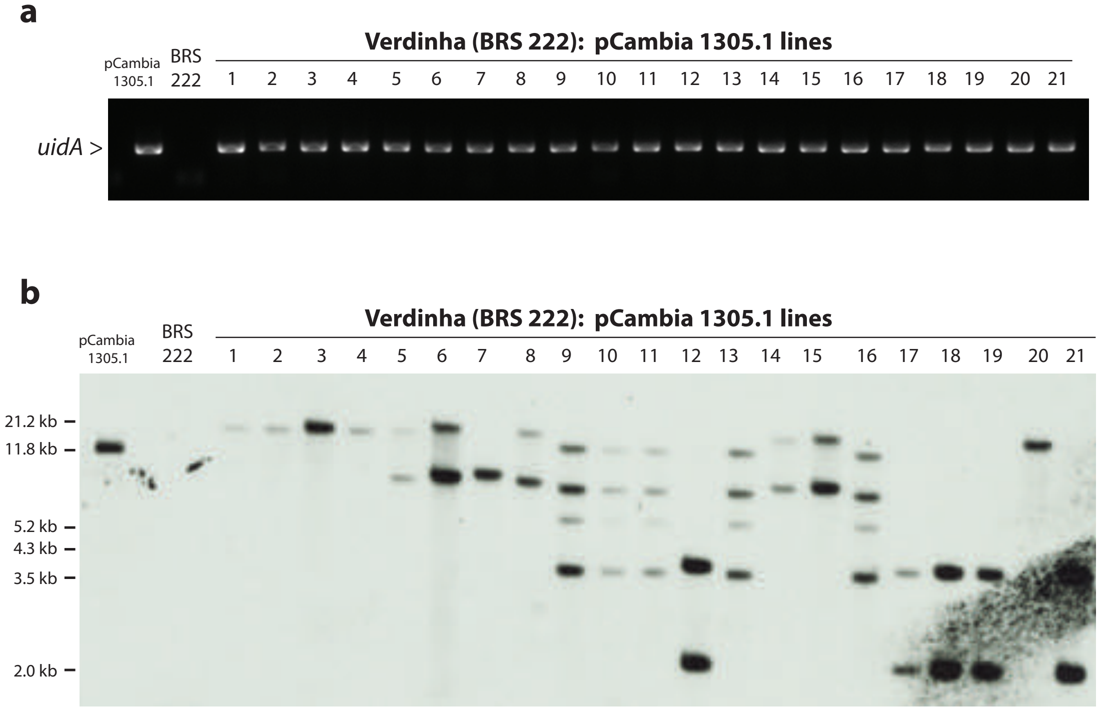

Figure S4 Phenotype of pCambia 1305.1-transgenic Verdinha (BRS 222) plants in greenhouse conditions. **(a)** Plantlets from different lines 1 month after rustication. **(b)** Transgenic BRS 222 plants (pCAMBIA1305.1, lines 3 and 7) and non-transgenic BRS 222 wild-type plants(control) 4 months after transfer to pot.

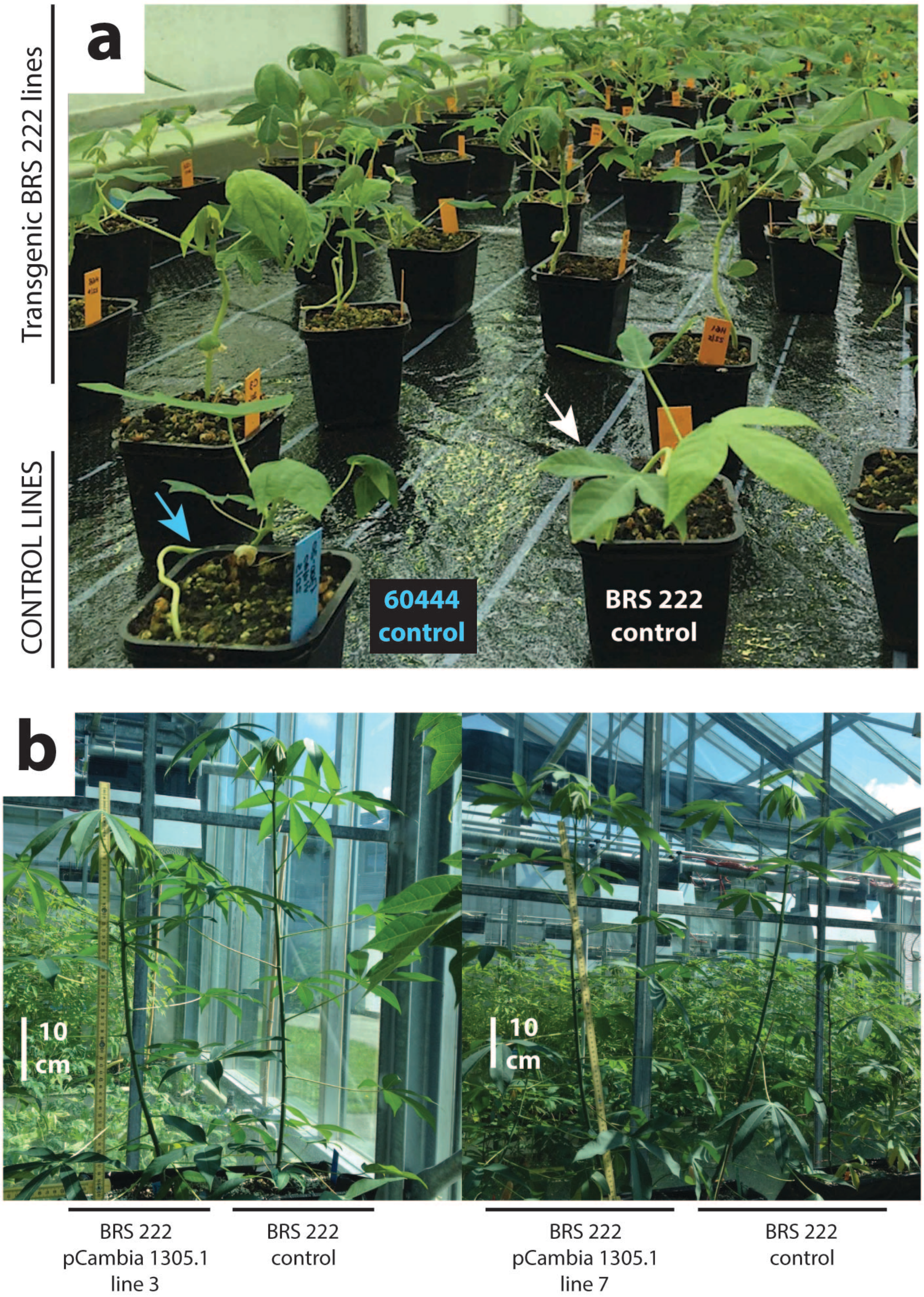

Supplement: Supplementary file 1 [file mps-01-00042-s001.pdf]
